# Fabrication of Gliadin–Carboxymethyl Chitosan Composite Nanoparticles to Improve the Stability and Antioxidant Activity of Curcumin

**DOI:** 10.3390/molecules30112414

**Published:** 2025-05-30

**Authors:** Xinyue Zhang, Mengdie Mo, Haiqi Yu, Hua Yang, Xu Liu, Yaping Xu, Xiaohui Zheng, Jie Wei, Fei Yu, Xiaodong Chen

**Affiliations:** 1 Medical College, Guangxi University, Nanning 530004, China; 2 Key Laboratory of Functional and Clinical Translational Medicine, Xiamen Medical College, Fujian Provincial University, Xiamen 361023, China; 3 College of Agriculture, Guangxi University, Nanning 530004, China; 4 Suzhou Key Lab of Green Chemical Engineering, School of Chemical and Environmental Engineering, College of Chemistry, Chemical Engineering and Materials Science, Soochow University, Suzhou 215123, China

**Keywords:** gliadin, curcumin, carboxymethyl chitosan, nanoparticles, antioxidant activity, food

## Abstract

The antisolvent precipitation method was employed to synthesize curcumin-loaded gliadin–carboxymethyl chitosan (CMCS) composite nanoparticles (GCC NPs). When the gliadin/CMCS weight ratio was 2:1, the GCC NPs with an ideal negative charge (−27.57 ± 1.07 mV) and the minimum particle size (184.13 ± 5.49 nm) were obtained. With the addition of CMCS, the encapsulation efficiency (EE) of Cur was markedly improved from 77.46 ± 1.54% to 93.88 ± 1.31%. Under various pH values and salt concentrations, the GCC NPs displayed excellent colloidal stability. Specifically, after encapsulation within the GCC NPs, the antioxidant activity of Cur was markedly improved. In ABTS and DPPH assays, the SC_50_ values of the GCC NPs were 4.98 ± 0.07 µg/mL and 9.86 ± 0.29 µg/mL, respectively. In summary, the GCC NPs would be an effective platform for the delivery of Cur in food and pharmaceutical preparations.

## 1. Introduction

**Figure 1:**
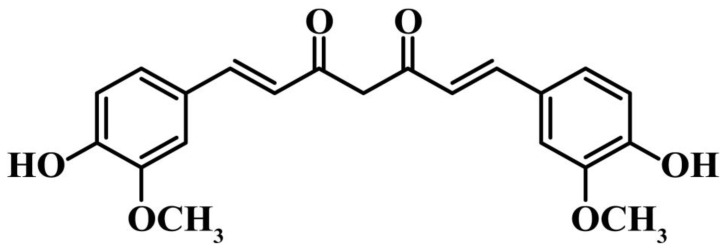
The chemical structure of Cur.

Curcumin (Cur), a natural plant polyphenolic diketone isolated from turmeric plants, is widely used in the food industry as a nutritional supplement (Figure 1) [1]. Meanwhile, many studies have been carried out on Cur in the biological and pharmacological fields due to its antioxidant and antimicrobial activities [2,3]. Nevertheless, the low solubility in aqueous solutions (<11 ng/mL), inherent physicochemical instability, and limited bioavailability of Cur have hindered its application in food and pharmaceutical systems [4]. Encapsulating Cur in protein-based nanoparticles to overcome these limitations has shown great potential in recent years [5]. Among these proteins, gliadin has attracted significant attention due to its excellent biodegradability and biocompatibility [6].

Gliadin, as an edible plant protein extracted from wheat, contains many hydrophobic amino acids and some hydrophilic amino acids, making it poorly soluble in water but easily soluble in 70% ethanol [7]. It has an excellent ability to form the nanoparticles, owing to its natural amphiphilic property and self-assembly capability [8]. Many reports have indicated that gliadin could be used to encapsulate various nutrients, including fucoxanthin and lutein [6,9]. However, gliadin nanoparticles are unstable and susceptible to forming aggregation due to the influence of the external environment, such as temperature and pH [9]. Some reports have found that the addition of hydrophilic polysaccharides could prevent the aggregation of gliadin by reducing the surface hydrophobicity of the nanoparticles [10].

Carboxymethyl chitosan (CMCS) is a chitosan derivative that dissolves in neutral aqueous solution [11]. It possesses many great characteristics, i.e., it is non-toxic, biocompatible, biodegradable, and negatively charged [12,13]. CMCS is often combined with positively charged proteins to form nanoparticles through the action of electrostatic interaction and hydrogen bonding, owing to its carboxyl group and multiple hydroxyl groups present in each unit [14,15]. Furthermore, its carboxylate anions could form the external hydrophilic shell of composite nanoparticles [16,17].

Recently, CMCS-stabilized gliadin nanoparticles have attracted wide attention due to their advantages of greater availability, application versatility, and economic potential [18]. These gliadin-based nanoparticles are usually prepared via the antisolvent precipitation method, which offers several advantages such as simplicity of preparation, high encapsulation efficiency, and avoidance of the extreme pH effects on bioactive compounds [19]. Zheng et al. have reported that a nanosystem based on gliadin and CMCS was developed to deliver coix seed oil (CSO), which improved its stability and bioavailability [15]. As far as we know, utilizing the antisolvent precipitation method to form CMCS-stabilized gliadin nanoparticles in Cur has never been reported.

**Figure 2:**
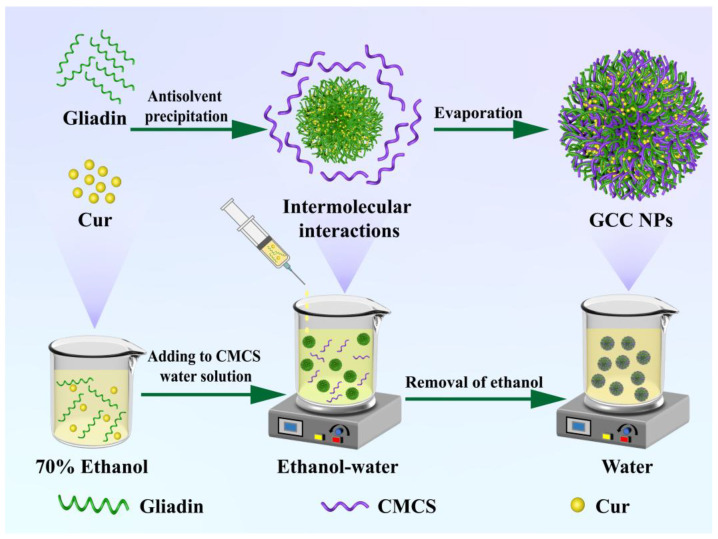
Diagram of synthesis mechanism of GCC NPs.

In this work, GCC NPs were self-assembled via antisolvent precipitation (Figure 2). The influence of CMCS concentrations on nanoparticles was investigated by measuring the particle size and zeta potential. Then, the characterizations of GCC NPs were performed using TEM, FTIR, and XRD. Moreover, the improvements in the encapsulation efficiency (EE), stability, and release properties of Cur in GCC NPs were assessed. Ultimately, the antioxidant activity of GCC NPs was also evaluated. This work may provide a basis for the interactions among gliadin, CMCS, and Cur, which could supply new insights for the development of nanosystems for delivering Cur in the food industry.

## 2. Results

### 2.1. Optimization of the Gliadin–CMCS NPs

**Figure 3:**
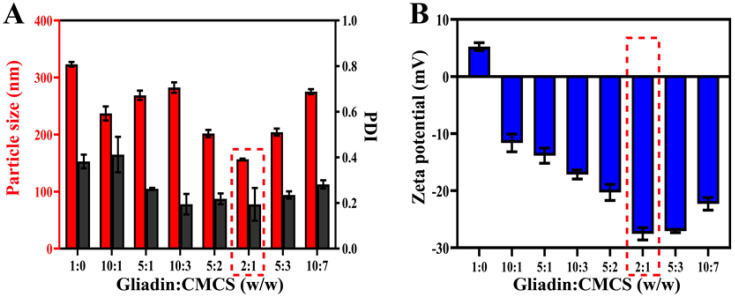
Effects of CMCS content on the (**A**) particle size, PDI, and (**B**) zeta potential of the gliadin–CMCS NPs. The red dotted box indicated the mass ratio that was selected for further studies.

Based on the antisolvent precipitation method, the influence of CMCS content on the physicochemical characteristics of the gliadin–CMCS NPs was examined. As can be seen from Figure 3A, the diameter of the gliadin NPs in the absence of CMCS was 323.10 ± 4.06 nm. When the mass ratios of gliadin to CMCS were changed from 10:1 to 5:2, the size of the gliadin–CMCS NPs showed varying degrees of decline. The reason for this phenomenon was that the electrostatic repulsion between the gliadin–CMCS NPs was enhanced owing to the incorporation of CMCS, which could prevent the aggregation of gliadin–CMCS NPs [3]. It should be noted that the particle size of the gliadin–CMCS NPs was the smallest when the mass ratio of gliadin to CMCS was 2:1 (Figure 3A). The results might be explained by the more compact structure of the gliadin–CMCS NPs after the addition of CMCS. However, the size of the gliadin–CMCS NPs gradually increased when the ratio rose from 2:1 to 10:7. The results were likely due to the excessive absorption of CMCS on the surface of gliadin, causing an increase in the size of the gliadin–CMCS NPs [20].

As presented in Figure 3B, when the mass ratio of gliadin to CMCS increased, the zeta potential of the gliadin–CMCS NPs changed from positive to negative, accompanied by a gradual escalation in the absolute value. Notably, the zeta potential (absolute value) reached the maximum at the ratio of 2:1. These findings demonstrated that the absolute value of the zeta potential was sufficiently high to ensure the electrostatic repulsion between the gliadin–CMCS NPs, which could hinder their aggregation. These results were consistent with those of a previous study which suggested that composite particles with high net charge could enhance their stability [21].

### 2.2. Characterization of the GCC NPs

**Figure 4:**
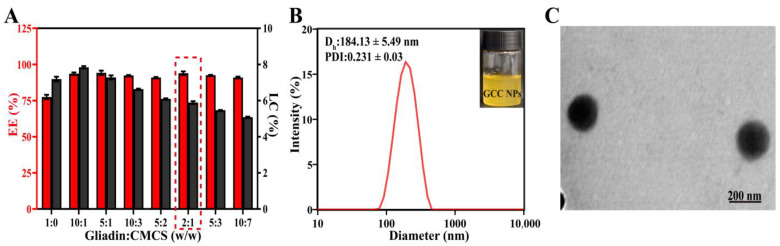
(**A**) Encapsulation efficiency (EE) and loading capacity (LC) of Cur loaded in the Cur-loaded gliadin–CMCS composite nanoparticles (GCC NPs). (**B**) Particle size distribution of the GCC NPs (inset: the visual appearance of the GCC NPs). (**C**) TEM image of the GCC NPs.

Next, the effect of CMCS content on the encapsulation efficiency (EE) and loading capacity (LC) of Cur in the GCC NPs was investigated. As illustrated in Figure 4A, the introduction of CMCS enhanced the EE of the GCC NPs compared to that of the GC NPs, which was consistent with previous reports [22]. When CMCS was incorporated, the EE of GCC NPs was markedly improved from 77.46 ± 1.54% to 93.88 ± 1.31%. The findings indicated that there was an enhancement effect of CMCS on the EE of Cur in the GCC NPs. At the mass ratio of 2:1, the LC was 5.89 ± 0.08%. Based on the above results, we chose 2:1 as the mass ratio of gliadin to CMCS in subsequent studies.

Meanwhile, the diameter of the GCC NPs at the mass ratio of 2:1 was 184.13 ± 5.49 nm (Figure 4B). Subsequently, the microstructure of the GCC NPs was visualized by TEM. As exhibited in Figure 4C, the GCC NPs showed well-defined regular spheres. The diameter of the GCC NPs in TEM was approximately 190 nm, which was consistent with the results measured by DLS.

### 2.3. FTIR, XRD, and DSC of GCC NPs

**Figure 5:**
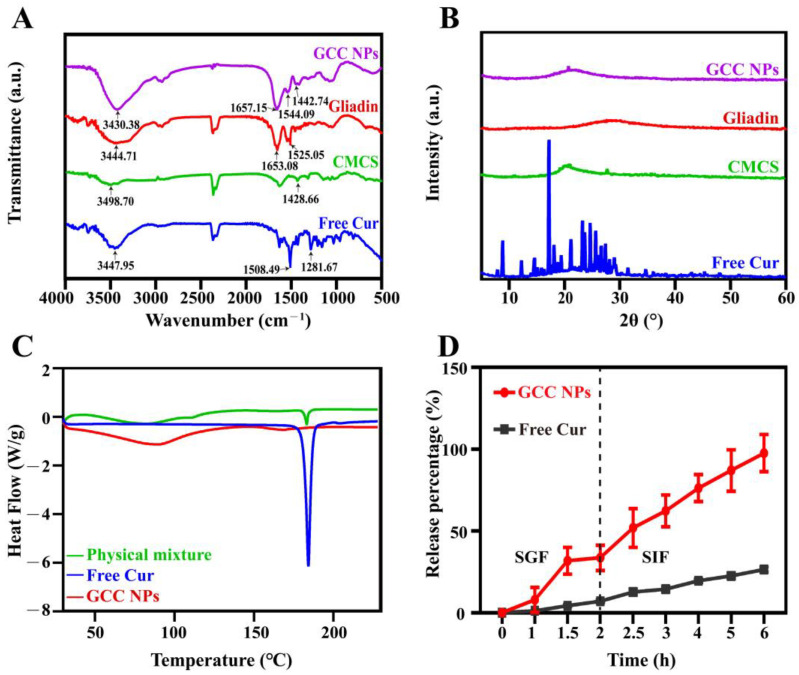
(**A**) FTIR spectra of free Cur, gliadin, CMCS, and GCC NPs. (**B**) XRD patterns of free Cur, CMCS, gliadin, and GCC NPs. (**C**) DSC profiles of free Cur, GCC NPs, and physical mixture. (**D**) Release profiles of free Cur and GCC NPs during simulated gastrointestinal digestion.

The FTIR spectra of the free Cur, gliadin, CMCS, and GCC NPs were presented in Figure 5A. In the spectrum of gliadin, the characteristic bands emerged at 1653.08 cm^−1^ and 1525.05 cm^−1^, which represented amide I and amide II bands, respectively [6]. Meanwhile, the characteristic band of CMCS (-COOH symmetrical stretching) emerged at 1428.66 cm^−1^ [20]. Notably, these bands in the GCC NPs moved to 1657.15 cm^−1^ (amide I band), 1544.09 cm^−1^ (amide II band), and 1442.74 cm^−1^ (-COOH), indicating that the intermolecular interactions (e.g., electrostatic interaction) might occur between gliadin and CMCS [15,23]. Additionally, most of the characteristic bands of free Cur (1281.67 cm^−1^ and 1508.49 cm^−1^) were not observed in the spectrum of the GCC NPs, suggesting the successful encapsulation of Cur, which was consistent with previous studies [3,24]. Furthermore, the characteristic bands of hydrogen bonding for gliadin, CMCS, and free Cur were presented at 3444.71 cm^−1^, 3498.70 cm^−1^, and 3447.95 cm^−1^, respectively. It should be noted that these O-H stretching bands were shifted to 3430.38 cm^−1^ in the GCC NP spectrum. Conclusively, all these findings indicated that hydrogen bonding and electrostatic interaction were dominantly involved in the formation of GCC NPs.

Since the changes in the crystalline state of Cur could influence its stability and solubility, the XRD patterns of the GCC NPs and free Cur were investigated. For free Cur, some characteristic diffraction peaks were observed at 8.79°, 12.27°, 14.57°, 17.19°, and 23.22°, which reflected its highly crystalline property (Figure 5B). Notably, these diffraction peaks of Cur did not exist in the XRD pattern of the GCC NPs, confirming that Cur was successfully encapsulated by the GCC NPs [25,26]. Moreover, the results also indicated that the Cur in the GCC NPs existed in an amorphous form, which could be attributed to the alterations in the molecular arrangement of Cur after being encapsulated in the GCC NPs [27]. In addition, these results could also be corroborated by DSC analysis, in which the sharp endothermic peak around 184.27 °C of Cur disappeared in the GCC NPs (Figure 5C) [28].

### 2.4. Cur Release in the Simulated Gastrointestinal Digestion

To estimate the release behavior of Cur, the GCC NPs were exposed to simulated gastrointestinal fluid. According to Figure 5D, the release rate of free Cur was 7.07 ± 0.70% in simulated gastric fluid (SGF) medium due to its limited solubility within gastric fluid. However, the cumulative release rate of the GCC NPs reached 33.63 ± 7.70% after 2 h of digestion in SGF. Subsequently, at the end of intestinal digestion, the cumulative release percentage of the GCC NPs reached 97.61 ± 11.35%, which was higher than that of free Cur (26.49 ± 1.57%). This was probably because the amorphous GCC NPs exhibited better water dispersity and release efficiency than that of free Cur (in crystalline form) [29]. Additionally, for the GCC NPs, it was discovered that the release rate of Cur from simulated intestinal fluid (SIF) was greater than that from SGF, which was consistent with previous report [2]. These observed phenomena can be primarily attributed to the fact that the electrostatic interaction between CMCS and gliadin in the GCC NPs was weakened in SIF. This weakness was attributed to the increased number of exposed gliadin digestion sites and the enhanced shear effect of trypsin in SIF [30]. The extent of Cur release from the GCC NPs was comparable to that of other Cur delivery systems [1], which would improve the absorption of Cur by the body.

### 2.5. pH Stability

**Figure 6:**
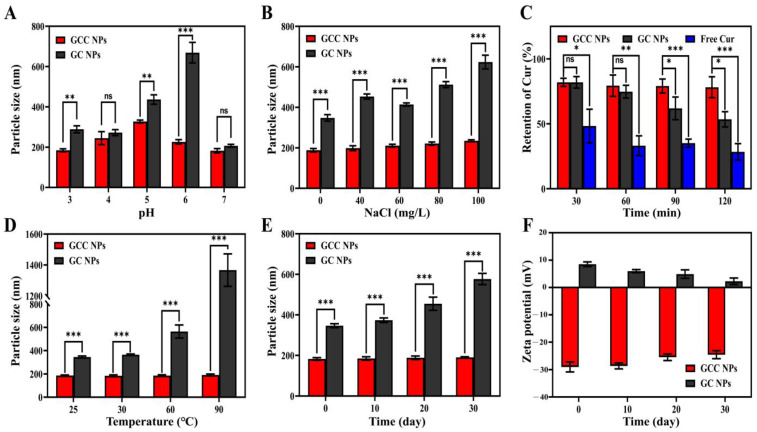
(**A**) Effect of pH on particle size of Cur-loaded gliadin–CMCS composite nanoparticles (GCC NPs) and Cur-loaded gliadin nanoparticles (GC NPs). (**B**) Effect of NaCl on particle size of GCC NPs and GC NPs. (**C**) Retention rate of Cur after UV light exposure. (**D**) Effect of heat treatment on particle size of GCC NPs and GC NPs. Effect of storage time on (**E**) particle size and (**F**) zeta potential of GCC NPs and GC NPs (ns: *p* > 0.05, * *p* < 0.05, ** *p* < 0.01, *** *p* < 0.001).

During the process of production, storage, and consumption, food products might be exposed to pH fluctuations. Apparently, the improvement in the stability of the GCC NPs to pH changes would facilitate their application in commercial products. Thus, it is necessary to investigate the impact of pH on GCC NPs. As exhibited in Figure 6A, the diameter of the GC NPs and the GCC NPs was evaluated across different pH conditions. Notably, the particle size of the GC NPs showed an obvious variation in response to changes in pH, which reached a maximum value of 668.83 ± 50.79 nm at pH 6. This pH was close to the isoelectric point of gliadin, which induced the weak electrostatic repulsions between the GC NPs and the high degree aggregation of the GC NPs [31]. In contrast, with the increase in pH from 3 to 7, the GCC NPs remained relatively stable. This phenomenon may be due to the fact that the steric repulsion between the hydrophilic polysaccharide chains could effectively stabilize the GCC NPs [32].

### 2.6. Ionic Strength Stability

The drug delivery system would be exposed to different salt ion strengths during the process of preparation and absorption in the human gastrointestinal tract. Therefore, it is critical to enhance the ionic strength stability of the GCC NPs. The diameter of the GC NPs and the GCC NPs in varying concentrations of NaCl solutions was measured to estimate the influence of salt ion strength on the nanoparticles (Figure 6B). When the NaCl concentration was increased from 0 to 100 mg/L, the diameter of the GC NPs significantly changed from 347.77 ± 16.08 nm to 623.70 ± 33.96 nm, which may be attributed to the electrostatic screening effect produced by the addition of NaCl [33]. In contrast, no obvious increase in particle size was observed for the GCC NPs (188.07 ± 8.76 nm to 234.60 ± 5.03 nm), indicating the outstanding salt resistance of the GCC NPs. The observed phenomenon could be attributed to the elevated surface electronegativity of the GCC NPs. This led to electrostatic repulsive forces, which were capable of mitigating the interference caused by NaCl [30]. These findings revealed that the GCC NPs exhibited better ionic strength stability compared to that of the GC NPs.

### 2.7. Photochemical and Thermal Stability

Cur is a highly photosensitive molecule, which limits its application. In this work, the photochemical stability of Cur in GCC NPs, free Cur and GC NPs was investigated under UV irradiation conditions. With the extension of exposure time, the retention rate of free Cur was sharply decreased. This was primarily attributed to the fact that Cur was susceptible to light (Figure 6C) [34]. In contrast, after the UV treatment for 30, 60, 90, and 120 min, the retention rates for Cur in the GCC NPs were 81.92 ± 3.12%, 79.36 ± 8.22%, 79.07 ± 5.38%, and 78.11 ± 8.09%, respectively. Notably, the Cur in the GCC NPs showed better stability than that in the GC NPs. Apparently, the synergistic effect of gliadin and CMCS provided the Cur with a more stable environment, protecting it [3]. In addition, the inherent UV-absorbing properties of conjugated double bonds and aromatic moieties within the gliadin molecule might account for the enhanced photostability of the GCC NPs [35]. These findings indicated that the GCC NPs could significantly enhance the stability of Cur against UV irradiation, which was in alignment with the previous report [36].

The thermal stability of each group was examined by incubating the groups in water baths at temperatures of 30, 60, and 90 °C for 30 min. According to Figure 6D, the GCC NPs and the GC NPs showed different sensitivity to heating. With the increase in temperature, the change of particle size of the GC NPs was more obvious than that of the GCC NPs. After heating at 90 °C for 30 min, the mean particle size of the GC NPs increased notably from 344.63 ± 9.17 nm to 1367 ± 104.70 nm. Conversely, the particle size of the GCC NPs remained basically unchanged as the temperature increased. Obviously, it could be concluded that coating the GCC NPs with CMCS impeded the aggregation triggered by heating.

### 2.8. Storage Stability

In commercial applications, it is critical to ensure storage stability. Therefore, the dimensional parameters and surface charge characteristics of the samples were quantified at various storage times. The average diameter of the GC NPs increased from 346.17 ± 10.06 nm to 576.77 ± 27.78 nm after 30 days of storage. In contrast, no obvious change in the diameter of the GCC NPs was observed (Figure 6E). Meanwhile, the zeta potential (absolute value) of the GCC NPs was consistently higher than 20 mV (Figure 6F). It was suggested that the addition of CMCS could improve the storage stability of the GCC NPs [37].

### 2.9. Antioxidant Activity

#### 2.9.1. DPPH and ABTS Radical Scavenging Capacity

**Figure 7:**
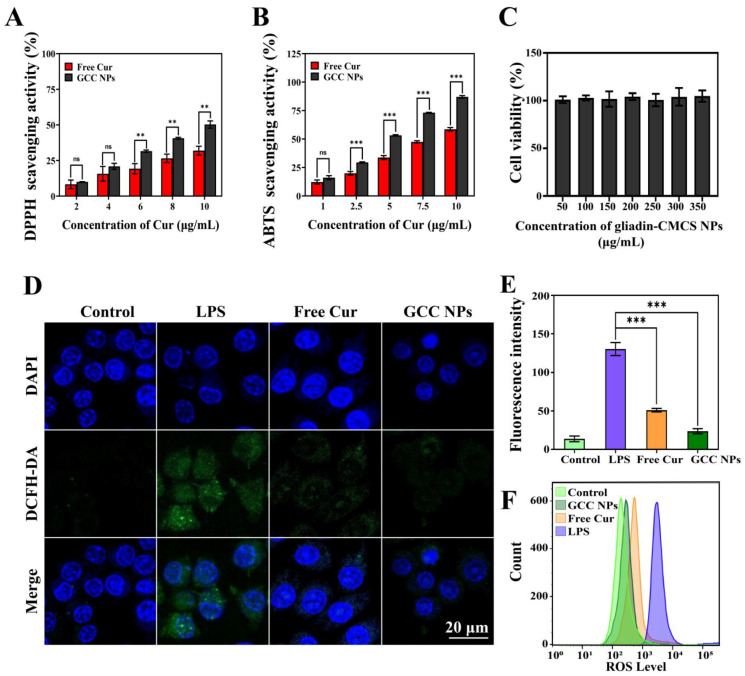
(**A**) DPPH and (**B**) ABTS radical scavenging ability of free Cur and GCC NPs. (**C**) Cytotoxicity against RAW 264.7 cells after incubation with gliadin–CMCS NPs for 24 h. (**D**) Confocal images of ROS-eliminating effects in RAW 264.7 cells. (**E**) Fluorescence intensity values of ROS in RAW 264.7 cells, calculated using ImageJ 1.53t software. (**F**) Flow cytometry analysis of ROS level in RAW 264.7 cells (ns: *p* > 0.05, ** *p* < 0.01, *** *p* < 0.001).

The antioxidant activity of the GCC NPs could be assessed through a DPPH radical scavenging assay. Cur could reduce the absorbance of DPPH because its phenolic hydroxyl groups could provide H+ ions to the DPPH radicals. As illustrated in Figure 7A, the DPPH radical scavenging activity of the free Cur and GCC NPs was positively correlated with the concentrations of all samples. At the concentration of 10 μg/mL, the GCC NPs exhibited better scavenging capacity (50.26 ± 2.54%) in the DPPH assay compared to that of the free Cur (31.92 ± 3.08%). Meanwhile, the SC_50_ of the GCC NPs and free Cur were 9.86 ± 0.29 μg/mL and 16.32 ± 1.47 μg/mL, respectively. The lower DPPH radical scavenging ability of free Cur was likely caused by its aggregation in water, which limited its interaction with the DPPH radicals [38]. However, this interaction could be enhanced after the hydrophobic Cur was loaded in the GCC NPs, which may be attributed to the improved dispersion of the GCC NPs [34]. On the other hand, many works suggested that CMCS may partly contribute to the improvement in the DPPH radical scavenging ability [39]. Based on these results, the GCC NPs possessed excellent capacity for scavenging DPPH radicals. Similarly, the GCC NPs also showed more efficient scavenging capacity in the ABTS measurement (Figure 7B). At 10 μg/mL, the ABTS radical scavenging rate of the GCC NPs reached to 87.02 ± 1.15%, which was higher than that of free Cur (58.59 ± 1.49%). This was consistent with the results for SC_50_ of the GCC NPs (4.98 ± 0.07 μg/mL) and free Cur (8.20 ± 0.22 μg/mL). Compared with previous reports, the GCC NPs showed a similar ability to scavenge radicals [40]. Overall, these experimental findings demonstrated that the GCC NPs could significantly enhance the free radical scavenging potential of Cur.

#### 2.9.2. Determination of Intracellular ROS

As illustrated in Figure 7C, no distinct cytotoxicity was detected in RAW 264.7 cells incubated with the gliadin–CMCS NPs. These results proved that the carrier in this work exhibited excellent biocompatibility. Therefore, the ROS scavenging capacity was further investigated. The overproduction and accumulation of ROS could lead to oxidative stress and cell damage. DCFH-DA, as a classical fluorescence probe, was utilized to measure the ROS level within the RAW 264.7 cells via CLSM. As can be seen in Figure 7D, intense green fluorescence was detected in the LPS-treated samples, reflecting an elevated production of ROS induced by LPS. Specifically, the cells treated with the GCC NPs exhibited notably weaker green fluorescence than that of other groups. This phenomenon indicated that a substantial amount of ROS had been removed from the cells. As displayed in Figure 7E, the fluorescence intensity of the ROS of each group in CLSM was quantified. It could be observed that the fluorescence intensity notably declined with the treatment of the GCC NPs. Moreover, the flow cytometry assay was also used to measure the ROS level in the cells (Figure 7F). The cells treated with the GCC NPs exhibited the lowest ROS level in comparison to other groups, which was consistent with the findings of the CLSM. The results demonstrated that the GCC NPs displayed better ROS scavenging ability in vitro.

## 3. Materials and Methods

### 3.1. Materials

Curcumin (purity > 97%), 2′,7′-dichlorodihydrofluorescein diacetate (DCFH-DA), and cell counting kit-8 (CCK-8) were obtained from Dalian Meilun Biotechnology Co., Ltd. (Dalian, China). Gliadin, CMCS, DPPH, ABTS, and potassium persulfate were purchased from Shanghai Macklin Biochemical Co., Ltd. (Shanghai, China). FBS was obtained from Cegrogen Biotech GmbH (Stadtallendorf, Germany). DMEM was acquired from Thermo Fisher Scientific Inc. (Waltham, MA, USA).

### 3.2. Preparation of Nanoparticles

The GCC NPs were formed via the antisolvent precipitation method [12]. Firstly, 100 mg of gliadin was dissolved in 10 mL of 70% (*v*/*v*) ethanol under magnetic stirring at 600 rpm for 1 h. Next, the mixture solution was centrifuged at 2000 rpm for 10 min at room temperature to remove insoluble residues. The concentration of gliadin in the supernatant was determined using a UV–Vis spectrophotometer. Subsequently, 10 mg of Cur was dissolved in the gliadin supernatant to acquire a gliadin–Cur solution. This solution (10 mL) was then rapidly added dropwise to 25 mL of CMCS aqueous solution under continuous stirring. To investigate the optimal mass ratio, the weight ratios of gliadin to CMCS were set as 1:0, 10:1, 5:1, 10:3, 5:2, 2:1, 5:3, and 10:7, respectively. The quality of CMCS was based on the gliadin content in the supernatant. The mixture was then stirred at 600 rpm for 1 h, and the ethanol was removed using a vacuum rotary evaporator to obtain the GCC NPs. For comparison, the Cur-loaded gliadin nanoparticles (GC NPs, without CMCS) were fabricated by dripping the 10 mL gliadin–Cur mixture into 25 mL of water, followed by the removal of ethanol, and the gliadin–CMCS NPs (blank NPs without Cur) were produced using the aforementioned methodology, except without the addition of Cur.

### 3.3. Particle Size, Polydispersity Index (PDI), and Zeta Potential

The hydrodynamic size, PDI, and zeta potential of the nanoparticles were analyzed using dynamic light scattering (DLS) (NANO ZS90, Malvern Instruments Ltd., Malvern, England). All test solutions were diluted 10-fold (*v*/*v*) with deionized water before measurement.

### 3.4. Transmission Electron Microscopy (TEM)

The morphological features of GCC NPs were obtained via TEM (HT-7700, HITACHI Co., Ltd., Tokyo, Japan). Before measurement, the freshly prepared sample was diluted with ultrapure water. Then, the above diluted sample was placed on a copper grid and left to dry naturally at room temperature.

### 3.5. Encapsulation Efficiency (EE) and Loading Capacity (LC)

Briefly, centrifugation was carried out on all samples for 30 min at 10,000 rpm. Afterward, the supernatant was gathered and diluted with an 80% (*v*/*v*) ethanol–water solution. Subsequently, the absorbance of the above solutions was determined by a UV–Vis spectrophotometer at 425 nm. A standard calibration curve was used to quantify the content of Cur. The EE and LC of GCC NPs were calculated as follows:

(1) $$\mathrm{EE}\;(\%)=\frac{Total\;Cur-free\;Cur}{Total\;Cur}\times 100$$

(2) $$\mathrm{LC}\;(\%)=\frac{Total\;Cur-free\;Cur}{Total\;amount\;of\;nanoparticles}\times 100$$

### 3.6. Fourier Transform Infrared Spectroscopy (FTIR)

The chemical structures of the pure Cur, gliadin, CMCS, and GCC NPs were investigated via an FTIR spectrophotometer (Nicolet iS10, Thermo Fisher Scientific Inc., Waltham, MA, USA). Pure KBr powder was mixed with each freeze-dried sample before being measured. After grinding, they were pressed into thin disks. Finally, a complete scan of the FTIR spectra was conducted, encompassing the wavenumber range from 500 to 4000 cm^−1^ in 64 scans with a 4 cm^−1^ resolution.

### 3.7. X-Ray Diffraction (XRD)

To investigate the crystalline structures of the GCC NPs, gliadin, CMCS and Cur, the XRD patterns of all samples were acquired via an X-ray diffractometer (A24A10, BRUKER AXS GMBH, Karlsruhe, Germany). The 2θ angle was set to 5–60°. The scanning rate was maintained at 10°/min.

### 3.8. Differential Scanning Calorimetry (DSC)

Thermal analysis of the free Cur, GCC NPs, and the physical mixture (including free Cur, gliadin, and CMCS) was conducted using DSC (DSA 25, TA Instruments, New Castle, DE, USA). Each freeze-dried sample (5.0 mg) was placed on an aluminum plate and sealed tightly. The samples were heated from 30 °C to 230 °C. A constant heating rate of 10 °C/min was employed. Dry nitrogen served as the carrier gas at a flow rate of 20 mL/min, while an empty aluminum plate was used as the reference for baseline measurements.

### 3.9. Simulated Gastrointestinal Digestion

The dialysis method was used to determine the release characteristics of the free Cur and GCC NPs [41]. Briefly, simulated gastric fluid (SGF) containing pepsin (3.2 mg/mL) was prepared. HCl was used to adjust the pH of SGF to 1.2. Simulated intestinal fluid (SIF) containing 2.0 mg/mL trypsin and 20 mg/mL bile salt was prepared, and the pH was adjusted to 7.5. Next, 2 mL of freshly prepared free Cur or GCC NPs was sealed in the dialysis bag (MWCO = 3500 Da) and placed in 20 mL of SGF. Each sample was shaken in an incubator at 37 °C for 2 h. After that, the dialysis bag was transferred into 20 mL of SIF for another 4 h. At designed time intervals (0, 1, 1.5, 2, 2.5, 3, 4, 5, and 6 h), the release medium (2 mL) was collected. The Cur content was quantified using a UV–Vis spectrophotometer via the method mentioned in Section 2.5.

### 3.10. Evaluation of the Stability of the Nanoparticles

#### 3.10.1. pH and Salt Stability

The impact of salt or pH on stability was evaluated by determining the particle size. The freshly prepared GC NPs and GCC NPs were diluted with ultrapure water. The pH of the GC NPs and GCC NPs was then adjusted to corresponding values (3–7) using 1 M NaOH or HCl. Different concentrations of NaCl solutions were prepared, ranging from 0 to 100 mg/L. Then, the GC NPs and GCC NPs dispersions were mixed with equal volumes of NaCl solution. The samples mentioned above were kept at 25 °C for 24 h before measurement.

#### 3.10.2. Photochemical Stability

The free Cur, GC NPs, and GCC NPs were freshly prepared. Then, they were placed in transparent containers. These samples were exposed to 254 nm UV light for 0.5, 1, 1.5, and 2 h. A total of 2 mL of samples were collected at each point. The remaining Cur content in the samples was quantified via UV–Vis spectrophotometer. The retention rate of the samples was calculated using the following equation:

(3) $$\mathrm{Retention}\;\mathrm{rate}\;\mathrm{of}\;\mathrm{Cur}\;(\%)=\frac{The\;remaining\;concentration\;of\;Cur}{The\;initial\;concentration\;of\;Cur}\times 100$$

#### 3.10.3. Thermal Stability

The freshly prepared GC NPs and GCC NPs were placed in test tubes. The test tubes were then placed in separate water baths at 30 °C, 60 °C, and 90 °C and heated for 30 min at each temperature. Subsequently, the nanoparticle dispersions were cooled down to ambient temperature. Finally, the diameter of the GC NPs and GCC NPs was determined.

#### 3.10.4. Long-Term Storage Stability

To assess the long-term storage stability of the GCC NPs, the nanoparticle dispersions were placed in the dark at 4 °C for 30 days. The zeta potential and particle size of the GCC NPs were detected every 10 days.

### 3.11. Antioxidant Activity Evaluation

#### 3.11.1. DPPH Radical Scavenging Activity

The DPPH radical scavenging capacity of the samples was tested, as previously described. Briefly, 0.1 M DPPH ethanol solution was freshly prepared. The samples were mixed with equivalent aliquots of the above solution. Subsequently, the prepared solutions were incubated in the dark for half an hour to ensure experimental consistency. The control group contained deionized water instead of the samples. Absorbance measurements were conducted using a spectrophotometer, with the detection wavelength set to 517 nm. The antioxidant potential of the samples against the DPPH radicals was quantitatively evaluated using the following equation:

(4) $$\mathrm{DPPH}\;\mathrm{scavenging}\;\mathrm{activity}\;(\%)=(1-\frac{As}{Ac})\times 100$$

where Ac was the control group absorbance, and As was the test sample absorbance.

#### 3.11.2. ABTS Radical Scavenging Activity

The GCC NPs and free Cur were tested for ABTS radical scavenging, as previously described. Firstly, a 7 mM ABTS reserve solution was prepared by dissolving the ABTS powder in deionized water. Subsequently, the ABTS radicals were generated by adding potassium persulfate (2.45 mM) to the prepared solution. After 16 h, the above reaction solution was diluted with ethanol. The ABTS working solution could be used when its absorbance value was adjusted to 0.70 ± 0.02 at 734 nm. The sample was mixed with the above working solution at a ratio of 1:2 (*v*/*v*). The mixture was incubated for 10 min. An ultraviolet spectrophotometer was then applied to detect the absorbance of the samples. The scavenging capability of the GCC NPs and free Cur was determined using the equation below:

(5) $$\mathrm{ABTS}\;\mathrm{scavenging}\;\mathrm{activity}\;(\%)=1-\frac{As}{Ac}\times 100$$

where Ac was the ABTS working solution absorbance, and As was the test sample absorbance.

### 3.12. Cell Viability Assay

Cellular viability was quantitatively determined using the CCK-8 colorimetric method. RAW 264.7 macrophages were plated at a concentration of 5000 cells per well in 96-well culture plates and incubated overnight. After 24 h of treatment with gliadin–CMCS NPs, 10% CCK-8 reagent was added to each well. The cells were then held at 37 °C for 30 min, and the absorbance at 450 nm was detected.

### 3.13. Determination of Intracellular Reactive Oxygen Species (ROS)

DCFH-DA was used to assess the ROS levels. After plating in 12-well plates or confocal dishes, RAW 264.7 cells were cultured overnight. LPS, at a concentration of 50 ng/mL, was administered to stimulate intracellular ROS production. The cells were then exposed to GCC NPs and free Cur at a Cur concentration of 10 μg/mL. Following 24 h of incubation, DCFH-DA (10 μM) was added to the cells. Subsequently, the cells were kept in the dark for 30 min. The fluorescence intensity of different groups was captured using a CLSM (Olympus, Tokyo, Japan) or a flow cytometer (Invitrogen, Carlsbad, CA, USA).

### 3.14. Statistical Analysis

All measurements were repeated at least in triplicate. The data were recorded and subsequently statistically analyzed to determine significant differences using Graph Pad Prism 9.5.0.

## 4. Conclusions

In this work, the GCC NPs were successfully produced via the antisolvent precipitation method to deliver Cur. When the weight ratio of gliadin to CMCS was 2:1, the GCC NPs exhibited the smallest particle size and an optimal negative charge. Furthermore, FTIR was used to verify that electrostatic attraction, and hydrogen bonding may be involved in the formation of the GCC NPs. XRD and DSC analyses confirmed the successful encapsulation of Cur in the GCC NPs. Meanwhile, the GCC NPs were found to greatly increase the release of Cur in simulated gastrointestinal fluids. The stability of the GCC NPs was also remarkably improved with the help of CMCS. Notably, the GCC NPs significantly enhanced the antioxidant activity of Cur compared to that of free Cur. The GCC NPs developed in this study offer a novel strategy for Cur delivery in functional foods and pharmaceutical products.

## Data Availability

The original contributions presented in this study are included in the article. Further inquiries can be directed to the corresponding author.

## Abbreviations

The following abbreviations are used in this manuscript:

Cur	curcumin
CMCS	carboxymethyl chitosan
GCC NPs	curcumin-loaded gliadin–CMCS composite nanoparticles
EE	encapsulation efficiency
LC	loading capacity
ABTS	2,2′-azino-bis (3-ethylbenzothiazoline-6-sulfonic acid)
DPPH	1,1-diphenyl-2-picrylhydrazyl
TEM	transmission electron microscopy
FTIR	Fourier transform infrared spectroscopy
XRD	X-ray diffraction
SIF	simulated intestinal fluids
SGF	simulated gastric fluids
DLS	dynamic light scattering
PDI	polydispersity index
SC_50_	concentration of sample able to scavenge 50% of radicals
CCK-8	cell counting kit-8
DCFH-DA	2′,7′-Dichlorodihydrofluorescein diacetate
